# Burnout Syndrome and Absenteeism Among Nursing Staff at a Secondary-Level Hospital in Western Mexico: A Gender-Based Cross-Sectional Analysis

**DOI:** 10.3390/nursrep16040123

**Published:** 2026-04-07

**Authors:** José Juan Gómez-Ramos, Maria Eloísa Pérez-Ruíz, Ingrid Patricia Dávalos-Rodríguez, Bernardo Alejandro Mata-Villafan, Jaime Jesús Antón-García, Noé Moisés Flores-Jiménez, Alejandro Marín-Medina

**Affiliations:** 1 Especialidad de Medicina de Urgencias, Adscrita al Centro Universitario de Ciencias de la Salud (CUCS), Universidad de Guadalajara, Guadalajara 44340, Jalisco, Mexico; josejuan79@yahoo.com (J.J.G.-R.); elo_0293@hotmail.com (M.E.P.-R.); 2 Departamento de Urgencias, Hospital General de Zona 89, Instituto Mexicano del Seguro Social (IMSS), Guadalajara 44100, Jalisco, Mexico; 3 Departamento de Biología Molecular y Genómicas, Centro Universitario de Ciencias de la Salud (CUCS), Universidad de Guadalajara, Guadalajara 44340, Jalisco, Mexico; ingriddavalos@hotmail.com; 4 Centro de Investigación Biomédica de Occidente (CIBO), Instituto Mexicano del Seguro Social (IMSS), Guadalajara 44340, Jalisco, Mexico; 5 Licenciatura en Médico Cirujano y Partero, Centro Universitario de Ciencias de la Salud (CUCS), Universidad de Guadalajara, Guadalajara 44340, Jalisco, Mexico; bernamata024@gmail.com (B.A.M.-V.); jaimejag1018@gmail.com (J.J.A.-G.); floresjimeneznoemoises14@gmail.com (N.M.F.-J.)

**Keywords:** burnout, absenteeism, nursing staff, hospital, gender analysis, occupational health

## Abstract

**Background**: Examining the relationship between burnout and absenteeism is important for understanding how chronic occupational stress translates into economic costs, reduced productivity, and deterioration in the health of nursing staff. The aim of this study was to evaluate absenteeism among nursing staff and its association with burnout from a gender perspective. **Methods**: A total of 154 nursing professionals with permanent contracts were included. An interview was conducted, which included the collection of sociodemographic data, characteristics related to their employment status, and the Maslach Burnout Inventory questionnaire in its Spanish-validated healthcare personnel version. The absenteeism rate was calculated using information from the hospital’s human resources department. The Mantel–Haenszel test was used to identify the association between burnout and absenteeism from a gender perspective. A *p*-value < 0.05 was considered statistically significant. **Results**: The prevalence of burnout was 70.1%; 52.6% reported absenteeism in 2024. The general nursing category was significantly associated with burnout (*p* = 0.039). Although no association was found between burnout and overall absenteeism, holding multiple jobs was identified as a determinant of partial absenteeism (*p* < 0.05). The hospital absenteeism rate was 4.8%. No statistically significant difference was found between burnout, gender, and absenteeism, with an adjusted odds ratio of 1.386 (95% CI: 0.75–2.65) after controlling for the effect of gender (*χ*^2^_MH_ = 0.672, *df* = 1, *p* = 0.412). **Conclusions**: Nursing staff present a critical level of burnout. No statistically significant difference was found between burnout, gender, and absenteeism, which could indicate that gender roles in the workforce may be changing in our population.

## 1. Introduction

Burnout is a psychological syndrome characterized by emotional exhaustion (EE), depersonalization (DE), and reduced personal accomplishment (PA). Its classification as a syndrome indicates that the symptoms collectively define a distinct clinical condition [1].

In 2019, the World Health Organization (WHO) included burnout syndrome in the International Classification of Diseases, 11th Revision (ICD-11) as an occupational phenomenon; that is, a factor that influences people’s health status and that may motivate them to seek medical attention. However, it is not classified as a disease or a specific health condition. According to the WHO, burnout is limited exclusively to the workplace, so its definition is not applicable to other areas of life [2].

Nursing staff play a vital role in the delivery of healthcare; however, they face complex tasks and difficult decisions in a stressful work environment, including long working hours, rotating shifts, high emotional demands [3,4], and, at times, verbal or physical aggression from patients, their families, or even colleagues [5]. Therefore, they are especially vulnerable to developing EE, DE, and reduced PA at work. These dimensions constitute the core components of burnout [6,7].

Among healthcare workers, there has been wide variability in the prevalence of burnout syndrome in low- and middle-income countries with rates ranging from 2.5% to 87.9%. In contrast, in the United States, studies evaluating burnout in physicians have reported prevalences as high as 67% [8]. High rates of burnout have also been reported among health profession educators [9].

Burnout syndrome among nursing staff has been widely studied, with an estimated global prevalence of 11%, although considerable regional variations exist [10]. The Asia-Pacific region has the highest prevalence, followed by Latin America and the Caribbean. Furthermore, a particularly high prevalence has been reported among nurses working in intensive care units [11].

In Mexico, the prevalence of burnout among nursing staff varies widely, with reports ranging from 33.8% to 91% [11,12]. Studies report the highest prevalence among nursing staff working in the emergency department (ED) and pediatric units [13,14].

When analyzing burnout syndrome from a gender perspective, earlier studies reported higher prevalence rates among women. However, more recent research has observed an increase in burnout rates among men, in some cases even exceeding those reported in women [15,16,17,18]. This increase has been associated with several factors, including marital status, having multiple children, job satisfaction, working night shifts, and employment in departments such as emergency medicine, psychiatry, or pediatrics [19].

Examining gender differences in burnout is important because it improves our understanding of the factors associated with its prevalence. This knowledge can also support the development of effective interventions aimed at improving the work environment and promoting more equitable working conditions [20].

Several studies have shown that individuals experiencing high levels of burnout exhibit higher rates of absenteeism [21]. Furthermore, despite progress in gender equality, substantial gaps in absenteeism rates persist, particularly among nursing staff [22].

Absenteeism represents a significant issue with substantial socioeconomic impact in hospital settings, as it affects the availability of human resources and the continuity of patient care, shifts the workload to other staff members, and contributes to increased staff turnover [23]. A year-long study conducted at a pediatric hospital in Mexico City estimated that the cost of absenteeism amounted to MXN 11,813,229.29 (approximately USD 670,380.21, based on the exchange rate at that time) [24]. These findings emphasize the urgent need to develop comprehensive workplace wellness programs to reduce absenteeism.

The influence of gender on the relationship between burnout and absenteeism among nursing staff has not been sufficiently explored. Despite efforts to understand these gaps, no single, conclusive explanation exists. In this context, the present study aims to analyze gender differences in burnout and examine how these disparities are associated with absenteeism among nursing staff. To this end, it integrates previous perspectives that highlight the influence of factors such as gender roles, job demands, and certain psychosocial characteristics.

## 2. Materials and Methods

### 2.1. Study Design and Setting

This was a cross-sectional correlational study. The study followed the STROBE guidelines. It was conducted at Hospital General de Zona No. 89 (HGZ 89) of the Instituto Mexicano del Seguro Social (IMSS), a secondary-level hospital located in the city of Guadalajara in western Mexico. The hospital has 226 registered beds and 137 unregistered beds.

### 2.2. Participant Selection

A total of 154 nurses participated in the study between September and November 2024. The study included nursing staff with permanent contracts at HGZ 89, comprising registered nurses, certified nursing assistants, clinical nurse specialists, and head nurses.

The sample size was calculated using the formula for a finite population, considering the total number of nursing staff assigned to HGZ 89 (*N* = 556), which included 293 registered nurses, 147 certified nursing assistants, 62 head nurses, and 54 clinical nurse specialists. Assuming a 95% confidence level and a 5% margin of error, the required sample size was estimated at 149 participants. The final sample size was *N* = 154, which met the minimum requirement and ensured adequate statistical power. All participants provided written informed consent. Burnout was considered the exposure variable, absenteeism the outcome, and sex was treated as a stratification variable.

### 2.3. Collection of Demographic and Employment Data

Participants were interviewed to collect sociodemographic data (age, sex, comorbidities, alcohol and tobacco use, family situation, and, where applicable, number of children) and aspects related to their employment status (contract category, work shift, work area, and years of seniority). Additionally, data on absenteeism in the last month were gathered, including absence, tardiness, and early release.

### 2.4. Assessment of Burnout Syndrome

Burnout status was assessed using the Maslach Burnout Inventory (MBI). The questionnaire measures the intensity and frequency of burnout. In this case, the validated version designed specifically for healthcare personnel in Spanish was used. This version consists of 22 items expressed as statements that explore feelings and attitudes toward work. Items were rated on a seven-point Likert scale (0 = never, 1 = a few times a year or less, 2 = once a month or less, 3 = a few times a month, 4 = once a week, 5 = a few times a week, 6 = every day) and were grouped into three dimensions: Emotional Exhaustion (EE), Depersonalization (DE), and Personal Accomplishment (PA). The test has an overall Cronbach’s alpha coefficient of 0.80; each subscale shows an alpha of 0.90 (EE), 0.79 (DE), and 0.71 (PA). The subscales are scored individually using the following cut-off points: for EE: 0–18, low; 19–26, medium; and 27, high. For DE: 5, low; 6–9, medium; and 10, high; for PA: 48, high; 34–39, medium; and 33, low. The prevalence of burnout was defined as a high score in at least one of the three dimensions; in the DE and EE dimensions, a higher score indicates greater exhaustion, while in the PA dimension, the opposite occurs [25].

### 2.5. Calculation of the Absenteeism Index

The absenteeism rate was calculated using records from the personnel department of HGZ 89. The absenteeism index was defined as the percentage of absence days (or hours) relative to the total contracted work time during 2024 [26].

### 2.6. Statistical Analysis

The IBM^®^ SPSS Statistics 24 software package was used for statistical analysis. Sociodemographic and clinical variables were analyzed using descriptive statistics (frequencies, means, and standard deviations) and tests of association (chi-square, Student’s *t*-test, Fisher’s exact test, and the Mann–Whitney U test). The selection between Student’s *t*-test and the Mann–Whitney U test was based on the assessment of normality using the Kolmogorov–Smirnov test. The Mantel–Haenszel test was performed to identify the association between burnout and absenteeism from a gender perspective. A *p*-value <0.05 was considered statistically significant.

### 2.7. Ethical Considerations

The study was carried out in accordance with the principles of the Declaration of Helsinki, and all study participants signed an informed consent document. The Local Ethics and Health Research Committee of HGZ 89 (1307) reviewed and approved the study protocol, with approval number R-2024-1307-060.

## 3. Results

### 3.1. Sociodemographic and Employment-Related Characteristics

Of the 154 participants, 62.3% were women, with a predominance of married (44.2%) and single (37.7%) individuals. A quarter of the participants had at least one comorbidity (25.3%), mainly hypertension (11.7%) and diabetes mellitus (DM) (7.1%). The prevalence of reported mental disorders was low. Sociodemographic characteristics are presented in Table 1.

Regarding their employment status, most participants were registered nurses (62.3%), and the majority worked in the emergency department (32.5%) and in the internal medicine unit (23.4%). As for their work shift, 45.5% worked the evening shift, and their average job tenure was 122.5 months (10.2 years). This information is presented in Table 2.

Regarding the relationship between burnout and sociodemographic and work-related variables, a statistically significant association was observed only between burnout and the category of registered nurse (*p* = 0.039), while no significant association was found with absenteeism. With respect to comorbidities, the presence of DM was significantly associated with absenteeism (*p* = 0.018). Widowed participants did not show a statistically significant association with burnout (*p* = 0.088), although their *p*-value was close to statistical significance. Regarding work variables, no statistically significant associations were observed.

### 3.2. Burnout and Absenteeism in Nursing Staff

An average burnout level of 58.9 points was found (Table 3), with an average score of 56.5 in men and 60.3 in women. The most severely impacted dimension was PA, whereas DE and EE scores remained relatively stable. It was also found that 36.4% of respondents exhibited high scores in at least one dimension; in 16 cases (10.4%), severe burnout was categorized in all three dimensions.

When analyzing sex differences among participants with burnout, a higher prevalence was observed in women than in men (63.9% vs. 36.1%), although this difference did not reach statistical significance. This pattern was more evident in the dimensions of EE and DE among women, whereas men showed higher scores in PA. Additionally, men exhibited higher absenteeism across its three forms: absence (8.6%), tardiness (35.8%), and early departure (35.8%).

When analyzing absenteeism, 33.1% of respondents reported having requested an early release in the last month, 28.5% reported tardiness, and only 11% reported work absence.

Finally, regarding the factors related to absenteeism (Table 4), the main factor associated with work absence was unjustified illness. Regarding tardiness, the most frequent causes were transportation problems and holding a second job. Early releases were mainly due to family matters, followed by holding a second job and childcare responsibilities.

Statistically significant differences were observed for holding a second job among participants who were tardiness or who obtained early release during the month prior to the study (*p* = 0.042 and *p* = 0.003, respectively). In both cases, the prevalence was higher in men than in women.

Regarding gender differences in the factors associated with absenteeism, caregiving for dependent family members and childcare responsibilities were more prevalent among women, whereas having another job—a statistically significant factor—was more frequent among men.

### 3.3. Relationship Between Burnout, Absenteeism, and Gender

To evaluate the association between burnout and absenteeism while controlling for gender, a stratified analysis using the Cochran–Mantel–Haenszel test was performed. The adjusted odds ratio (OR = 1.386; 95% CI: 0.75–2.65) indicated no statistically significant association between burnout and absenteeism after adjustment for gender (*X*^2^MH = 0.672, df = 1, *p* = 0.412). The homogeneity of the association across sex strata was assessed using the Breslow–Day test, which was not statistically significant (*X*^2^ = 0.604, df = 1, *p* = 0.437). This result suggests that the association between burnout and absenteeism does not differ significantly between male and female nursing staff. Nevertheless, sex-based differences were observed in the factors associated with absenteeism: caregiving for dependent relatives and childcare responsibilities were more prevalent among women, whereas having a second job was more frequent among men.

### 3.4. Absenteeism Index Among Nursing Staff

The overall absenteeism index at HGZ 89 during 2024 was 3.3%, while for nursing staff, the rate was 4.8%.

## 4. Discussion

The influence of gender-related factors on the relationship between burnout and absenteeism among nursing staff remains largely unexplored; despite efforts to bridge these gaps, no single, definitive explanation has been established. This study aims to analyze gender differences in burnout and examine how these disparities relate to absenteeism among nursing staff. It integrates previous perspectives highlighting the role of gender roles, job demands, and psychosocial characteristics.

Regarding the sociodemographic characteristics of our population, we observed that the gender ratio among nursing staff was 1:1.5 (men to women, respectively). Although nursing continues to be considered a predominantly female profession in most countries, this situation appears to be changing, not only in Mexico. Some countries, such as the United States, Singapore, Tanzania, New Zealand, the United Kingdom, and Australia [27], have been reporting this trend since the last decade. In response to the global shortage of nursing personnel, healthcare systems have increasingly recruited men to expand the workforce. This shift has been facilitated largely by changing perceptions of the qualities required for caregiving roles in a profession historically associated with gender, particularly as the role of women in the labor market and that of men in contemporary nursing continue to be re-evaluated [28].

Our study observed a statistically significant relationship between burnout and being a registered nurse (*p* = 0.039). It is known that, in the hospital setting, nursing staff are among the professionals most affected by this syndrome [19], particularly those whose duties involve direct contact with patients. Furthermore, this finding could also be related to the rotating nature of general nurses in our population, in contrast to specialists, who tend to have a fixed workplace. Other studies have reported this relationship [29].

The prevalence of burnout syndrome identified in our population reached 70%, a significantly high figure that exceeds most findings reported internationally [10,30,31], regionally [10], and in Mexico [11,32,33]. However, it is important to acknowledge the existence of some national studies that do document prevalences in similar ranges [12,20].

While it is a fact that the level of burnout experienced by healthcare personnel, and especially by nursing staff, is alarming globally, the diagnosis of this condition currently poses a methodological challenge: there is no uniformity in diagnostic criteria nor a single validated and standardized tool for its measurement. This lack of consensus and the inherent variability of the instruments used may explain the wide disparity between the levels of burnout reported in the literature and suggests that the figure obtained in our study should be interpreted with caution, given the variable nature of its diagnosis.

In our study, the most affected dimension of burnout syndrome was EE. A greater tendency toward emotional and physical exhaustion related to paid work was observed among women, a phenomenon frequently associated with the social perception of the female role. This tendency could be linked to factors such as the burden of domestic responsibilities, the expectation of fulfilling traditional gender roles, and the lack of family recognition of women’s paid work [34]. It has also been reported that women with a “progressive” work ideology exhibit burnout rates similar to those of men, whereas women with a “traditional” ideology report significantly higher rates [35]. Therefore, in our population, there appears to be a convergence in which women show burnout rates similar to those of men, a phenomenon mainly observed in countries with a high quality of life [36].

Unlike previous studies [37], our findings indicate that burnout levels were higher among men, although this difference was not statistically significant. This atypical result could be influenced by cultural perceptions of the nursing profession in Latin America, where it is often associated with gender stereotypes and perceived as underpaid [38], which could affect men’s sense of professional success and job satisfaction. This context would explain why men with multiple jobs had a higher number of partial absences (*p* < 0.05).

In contrast to the general trends reported in the literature, in our study, absenteeism was more prevalent among men, especially those who were married and worked in the ED and the internal medicine units. As previously mentioned, these occupational and sociodemographic characteristics are recognized as predisposing factors for both burnout syndrome and absenteeism. Furthermore, statistical significance was observed in cases with comorbidities, specifically DM.

An important finding that should be considered a possible aggravating or confounding factor in the relationship between burnout and absenteeism is the presence of comorbidities. The significant association between DM and absenteeism (*p* = 0.018) highlights the multifactorial nature of work absence, suggesting a bidirectional relationship: chronic illness may exacerbate burnout, while work-related stress and prolonged hours are known risk factors for metabolic and cardiovascular diseases [39,40].

In addition to comorbidities, some studies indicate that the workload experienced, combined with the resulting physical and mental fatigue, contributes to high rates of absenteeism [32,33]. In the case of women, another factor that frequently contributes to absenteeism is the shortage of nursing staff, a common situation in low- and middle-income countries [34].

However, beyond the individual level, absenteeism in nursing transcends the individual and constitutes a critical problem for healthcare systems, as it compromises patient safety, increases hospital mortality, and deteriorates the quality of care. Furthermore, it generates excessive workload for the remaining staff, fueling a cycle of burnout, dissatisfaction, and staff turnover [41].

In our study, we found a significant association with two specific categories of absenteeism: tardiness and early release. In both cases, this association was observed with nursing staff who reported having a second job (multiple employment). Some studies indicate that work overload, combined with the resulting physical and mental fatigue, contributes to higher rates of absenteeism [32,33]. Another factor contributing to absenteeism, primarily among women, is the shortage of nursing staff, a situation commonly observed in low- and middle-income countries [34].

This situation is further aggravated by its clinical and operational consequences, generating additional costs related to overtime, staff replacement, and productivity loss. Al Menji H, et al. [41] reported that, in some low- and middle-income countries, the annual cost associated with nursing staff absenteeism can reach millions of dollars, with estimates as high as one billion dollars per year. These figures indicate that absenteeism not only affects continuity of care but also poses a significant financial threat to the sustainability of healthcare organizations.

Taken together, the combined effect of burnout, comorbidities, and absenteeism erodes institutional human capital, compromising the achievement of care objectives and operational efficiency and jeopardizing the long-term sustainability of healthcare services [41]. Ultimately, our findings underscore the need to address the well-being of nursing staff as a strategic component of patient safety and the stability of the healthcare system as a whole.

Among the work-related factors associated with absenteeism identified in this study, multiple jobholding stands out. In our study, the statistically significant association with absenteeism was concentrated in two specific modalities: tardiness and early release. In both cases, this significance was observed when nursing staff were multiple jobholders. Multiple jobholding, defined as holding a second job in addition to full-time employment [42,43,44,45], is common among nursing staff, particularly in health systems of emerging economies. From an economic and organizational perspective, it often reflects the need to supplement income due to insufficient wages, job insecurity, or the pursuit of additional skills [43,44,45]. Beyond individual labor supply, this phenomenon may also reflect structural deficiencies in human resource management from a governance perspective [44].

Beyond its underlying causes, multiple jobholding directly impacts an employee’s overall well-being. While multiple jobs can sometimes represent an individual strategy for economic stability, several studies indicate that multiple jobholding is associated with a greater overall workload, sleep deprivation, accumulated fatigue, and an impaired work–life balance—factors that contribute to the development of EE [43]. This condition can impair job performance, reduce productivity, and increase the risk of errors, which is especially critical in clinical settings where patient safety depends on continuous, high-quality care [43,44]. At the organizational level, multiple jobholding is associated with job dissatisfaction, lower institutional commitment, a higher probability of staff turnover, and professional disengagement behaviors (including partial or total absenteeism) [42,43]. Furthermore, when professionals work in institutions within the same sector, conflicts of interest and difficulties in shift planning can arise, complicating human resource management [43]. Therefore, multiple jobholding should be considered an indicator of the structural conditions of labor systems and human resource policies. Proper management of this phenomenon is essential to prevent burnout and ensure continuity and quality of care [43,44,45].

In the Mexican context, multiple jobholding is legally permissible, provided that there is no explicit contractual prohibition or conflict of interest. In the private sector, labor regulations generally prioritize freedom of employment and contractual autonomy, whereas the public sector requires compliance with schedule compatibility and the efficient performance of duties to ensure the appropriate use of public resources [46]. From this perspective, healthcare organizations may require regulatory compatibility assessments to prevent service disruptions and ensure transparency in the performance of both roles. Currently, within the IMSS, multiple jobholding within the institution is only regulated through the allocation of overtime hours. This regulation is established in the collective bargaining agreement [47], the Social Security Law [48], and the procedure for authorizing overtime work [49] and is aligned with the provisions of the Federal Labor Law [50], which stipulates that overtime hours should not exceed 9 hours per week or 20 h per two-week period.

When analyzing the relationship between burnout and absenteeism from a gender perspective, we found no statistically significant difference, suggesting the absence of a significant association between burnout and absenteeism after controlling for the effect of gender (*X*^2^MH = 0.672, df = 1, *p* = 0.412). This finding may be interpreted in the context of evolving gender roles in the workplace, particularly in the nursing profession. As men and women enter different occupations, the stereotypes associated with these roles also change. Theoretical evidence suggests that repeated exposure to new, non-stereotypical role models can play a crucial role in reversing the traditional image of nurses [17,36,37]. Therefore, the absence of a significant difference in our population could indicate that roles and responses to work-related stress are becoming more homogenized between genders in this professional setting. For example, in developing countries, women must balance the responsibilities of paid employment with the demands of the home and family care, while men often seek opportunities to supplement their income and support their families. Both dynamics, driven by traditional gender roles, have been consistently associated with increased absenteeism [17].

From this perspective of homogenization, the impact of burnout and absenteeism should be analyzed not only as an individual phenomenon but also as a response to structural deficiencies in personnel management. The high prevalence of burnout detected (70.1%) in our sample suggests that current interventions are insufficient; therefore, a shift is needed from a traditional administrative approach to evidence-based strategic management, oriented toward institutional policies that prioritize job satisfaction, as well as epidemiological surveillance and close monitoring of comorbidities.

The increasing prevalence of burnout syndrome is causing a structural and economic crisis in healthcare services, as it increases absenteeism and staff turnover [51]. A study conducted in a Latvian hospital revealed that 30% of nursing staff intended to leave their jobs due to factors related to burnout syndrome [52]. This severely affects staff management, increases turnover, and, in the long term, has a profound impact on healthcare. In this regard, some studies have analyzed how policies designed to improve certain aspects of the hospital’s physical and social environment can improve the well-being and job satisfaction of nursing staff and decrease the prevalence of burnout [53]. Consequently, some countries have designed or re-evaluated occupational health policies related to burnout, such as promoting healthy work environments and providing psychological support in developing coping skills, self-care, and personal development [54]. It is also important to note that, while programs designed to reduce the prevalence of burnout syndrome exist, their dissemination among healthcare personnel has not been sufficient. For example, a multicenter study conducted in China revealed that only 35.9% of participants were aware of such programs, while 50% reported a lack of access to mental health resources in their workplace [18]. Therefore, it is necessary to strengthen the dissemination of occupational health programs aimed at reducing the prevalence of burnout, as well as to develop interventions tailored to the specific needs of each population, taking into account changes in gender roles. In this regard, the results observed in our study are consistent with the models proposed by Iverson et al. in 1988 and 2001 [55] regarding absenteeism. On the one hand, the hypothetical model of absenteeism suggests that absenteeism among healthcare personnel is associated with burnout and that this relationship is influenced by factors such as stress and job demands. In our study, these factors are reflected in two variables: the role of registered nurse and the presence of DM, both of which can increase burnout, as they are associated with both the demands inherent to the professional role and the burden derived from self-care. On the other hand, the affective model of absenteeism [56] proposes that withdrawal behaviors—including absenteeism, tardiness, and early release (the latter observed significantly in our sample)—function as defense mechanisms in response to an unsatisfactory and economically inadequate work environment, where job satisfaction is considered a key moderating variable.

These dynamics have direct implications for occupational health strategies in secondary-level hospitals. Likewise, they compel healthcare organizations to develop human resources policies that integrate general well-being programs and interventions aimed at improving work-related outcomes, such as productivity, work capacity, and absenteeism [57]. The available evidence suggests that organizations should implement targeted interventions, with programs aimed at high-risk subgroups. These strategies are more cost-effective than general policies [57,58]. Such measures would not only reduce the operating costs associated with absenteeism but also strengthen organizational support. In this context, it has been observed that, in managing absenteeism, self-care-based interventions (short-duration physical activity and personalized mental health counseling) have shown the best results, not only in reducing absenteeism but also in improving the work capacity index [58].

Specifically, in the case of DM, multiple interventions have been evaluated, most of them focused on self-management and self-care. In addition to the implementation of individualized counseling programs (virtual or in-person) [57], other interventions that have proven effective include granting time incentives (administrative leave) for self-care activities and the use of technological tools for metabolic monitoring. These strategies have demonstrated effectiveness in mitigating retirement behaviors associated with DM [57,59], improving the capacity for metabolic self-management without disrupting work schedules, and reducing lost productivity and the operating costs associated with staff replacement [57,58,59].

In Mexico, controlling unjustified absences is considered a critical indicator of organizational health. In 2020, the Mexican Social Security Institute (IMSS) adapted its internal policies to a strategic model of predictive and preventive management through the development of the “Servicios de Prevención y Promoción de la Salud para Trabajadores IMSS” (SPPSTIMSS by its Spanish acronym). This program established a national target of 3.1% for controlling unjustified absences and implemented the National Comprehensive System for Unjustified Absences, which identified worker health, the work environment, and the management of temporary disabilities as the main contributing factors.

However, the implementation of these strategies faces significant structural challenges. Although the SPPSTIMSS program has an allocated budget and a multidisciplinary team led by an occupational physician responsible for the evaluation and periodic monitoring of staff health [49], the high prevalence of burnout and its association with general nursing staff and those diagnosed with DM, as well as the impact of absenteeism observed among workers with multiple jobs (a situation reported in our study), suggest a possible disconnect between the regulatory framework and operational effectiveness in secondary care hospitals. In Mexico, information on the strategic management of nursing staff is limited, particularly regarding performance evaluation and the results of the SPPSTIMSS program. In this context, our study provides relevant empirical evidence supporting the transition to self-care and metabolic self-management models. Furthermore, our findings establish a solid foundation for auditing and redesigning existing occupational health programs from a sustainability perspective, with a greater emphasis on promoting the genuine well-being of healthcare workers.

Our study has limitations that should be considered when interpreting the results. First, the cross-sectional nature of its design prevents us from establishing definitive causal relationships between burnout, absenteeism, and gender. Similarly, since this study was conducted in a single secondary-level hospital, the generalization of the results to other hospital settings or regions should be approached with caution. Furthermore, the determination of absenteeism types (absences, tardiness, and time-outs) was based on participants’ self-reporting, which introduces a potential recall bias or social desirability bias, as it was not directly cross-referenced with official personnel records. Finally, since the prevalence of burnout syndrome was defined based on the presence of at least one of its three dimensions, the prevalence rate may have been overestimated.

To mitigate the high prevalence of burnout and absenteeism among our staff, we propose five key improvement strategies: 1. Optimize workload by adjusting staffing levels to patient care needs, prioritizing high-demand areas such as emergency and internal medicine. 2. Implement stress management programs to reduce EE among general nursing staff. 3. Address multiple job holdings by considering salary reviews or compensatory measures that may reduce the need for a second job. 4. Promote workplace health by developing programs for the detection and management of chronic comorbidities, such as DM. 5. Develop flexible, gender-sensitive work policies that recognize sociocultural demands (e.g., support for women with caregiving responsibilities and for men who hold multiple jobs).

## 5. Conclusions

Our study observed an association between registered nursing and burnout. No significant association was identified between burnout and absenteeism after controlling for gender. Although burnout appeared to be more frequent among nurses, this difference did not reach statistical significance. Consequently, gender does not appear to be associated with burnout among nursing staff in our population. These findings are consistent with recent evidence suggesting that burnout rates among women and men are becoming increasingly similar, which may reflect changes in gender roles in the workplace. Therefore, it is essential that hospital human resources management develop strategies to reduce absenteeism, taking into account current transformations in gender roles and specific burnout risk profiles. Furthermore, researchers should conduct longitudinal studies with larger cohorts to better understand the behavior of burnout syndrome among nursing staff.

## Figures and Tables

**Table 1:** Sociodemographic characteristics of the participants.

Variable	General Value (*n* = 154)	Burnout		Absenteeism	
		Value (*n* = 108)	*p*	Value (*n* = 81)	*p*
Age	37.7 (±7.6)	37.3 (±8.4)	0.727 ^a^	36.9 (±6.8)	0.405 ^a^
Sex					
Women	96 (62.3)	68 (63.0)	0.618 ^b^	36 (44.4)	0.099 ^b^
Men	58 (37.7)	40 (37.0)	0.618 ^b^	45 (55.6)	0.099 ^b^
Marital Status					
Single	58 (37.7)	44 (40.7)	0.227 ^b^	30 (37.0)	0.866 ^b^
Married	68 (44.2)	49 (45.4)	0.642 ^b^	38 (46.9)	0.468 ^b^
Divorced	11 (7.1)	7 (6.5)	0.734 ^c^	4 (4.9)	0.263 ^b^
Cohabitation	15 (9.7)	8 (7.4)	0.147 ^c^	8 (9.9)	0.962 ^b^
Widowed	2 (1.3)	0 (0.00)	0.088 ^c^	1 (1.2)	1.000 ^c^
Comorbidities	39 (25.3)	30 (27.8)	0.733 ^b^	15 (18.5)	**0.041 ^b^**
Hypertension	18 (11.7)	12 (11.1)	0.845 ^b^	8 (9.9)	0.461 ^b^
Diabetes Mellitus	11 (7.1)	8 (7.4)	1.000 ^c^	2 (2.5)	**0.018 ^b^**
Depression	4 (2.6)	2 (1.9)	0.583 ^c^	3 (3.7)	0.622 ^c^
Epilepsy	4 (2.6)	4 (3.7)	0.318 ^c^	1 (1.2)	0.346 ^c^
Dyslipidemia	5 (3.2)	5 (4.6)	0.323 ^c^	1 (1.2)	0.191 ^c^
Asthma	9 (5.8)	8 (7.4)	0.281 ^c^	3 (3.7)	0.309 ^c^
Anxiety	9 (5.8)	7 (6.5)	0.725 ^c^	5 (6.2)	1.000 ^c^
Alcohol consumption	14 (9.1)	12 (11.1)	0.232 ^c^	8 (9.9)	0.721 ^b^
Tobacco use	40 (26.0)	29 (26.9)	0.703 ^b^	21 (25.9)	0.989 ^b^
Parental status	91 (59.1)	59 (54.6)	0.084 ^b^	45 (55.6)	0.347 ^b^
Number of children	1.25 (±1.25)	1.39 (±1.14)	0.373 ^a^	1.32 (±1.27)	0.562 ^a^

Note. Categorical variables are expressed as frequency (*n*) and percentage (%). Continuous variables are expressed as mean and standard deviation. ^a^ Mann–Whitney U test. ^b^ Pearson’s chi-square test. ^c^ Fisher’s exact test. Significant values in bold (*p* < 0.05).

**Table 2:** Characteristics related to the employment situation of the participants.

Variable	General Value (*n* = 154)	Burnout		Absenteeism	
		Value (*n* = 108)	*p*	Value (*n* = 81)	*p*
Category					
Head Nurse	6 (3.9)	4 (3.7)	1.000 ^c^	2 (2.5)	0.423 ^c^
Certified Nursing Assistant	37 (24.0)	22 (20.4)	0.104 ^b^	17 (21.0)	0.353 ^b^
Registered Nurse	96 (62.3)	73 (67.6)	0.039 ^b^	55 (67.9)	0.133 ^b^
Clinical Nurse Specialist	15 (9.7)	9 (8.3)	0.383 ^c^	7 (8.6)	0.628 ^b^
Work Area					
Adult Emergency Department	50 (32.5)	36 (33.3)	0.725 ^b^	26 (32.1)	0.918 ^b^
Pediatric Emergency Department	11 (7.1)	10 (9.3)	0.175 ^c^	5 (6.2)	0.622 ^b^
Pediatric Unit	5 (3.2)	3 (2.8)	0.754 ^b^	2 (2.5)	0.431 ^b^
Internal Medicine Unit	36 (23.4)	26 (24.1)	0.635 ^c^	21 (25.9)	0.668 ^c^
Geriatric Unit	13 (8.4)	9 (8.3)	1.000 ^c^	5 (6.2)	0.286 ^b^
General Surgery Unit	5 (3.2)	5 (4.6)	0.323 ^c^	4 (4.9)	1.000 ^c^
Traumatology Unit	5 (3.2)	2 (1.9)	0.158 ^c^	3 (3.7)	1.370 ^c^
Gynecology Unit	5 (3.2)	4 (3.7)	1.000 ^c^	4 (4.9)	0.191 ^c^
Operating Rooms	9 (5.8)	6 (5.6)	1.000 ^c^	4 (4.9)	0.736 ^b^
Administrative Offices	0 (0.0)	0 (0.0)	-	0 (0.0)	-
Outpatient Clinic	9 (5.8)	4 (3.7)	0.128 ^c^	5 (6.2)	1.000 ^c^
Gynecology Emergency Department	2 (1.3)	1 (0.9)	0.510 ^c^	2 (2.5)	0.498 ^c^
Obstetrics and Gynecology Unit	4 (2.6)	2 (1.9)	0.583 ^b^	3 (3.7)	0.622 ^c^
Work shift					
Day Shift	50 (32.5)	35 (32.4)	0.981 ^b^	26 (32.1)	0.918 ^b^
Evening Shift	70 (45.5)	46 (42.6)	0.274 ^b^	39 (48.1)	0.479 ^b^
Night Shift	34 (22.1)	27 (25.0)	0.180 ^b^	16 (19.8)	0.464 ^b^
Job tenure	122.5 (±83.2)	129.7 (±88.9)	0.490 ^a^	128.4 (±91.4)	0.179 ^a^

Categorical variables are expressed as frequency (*n*) and percentage (%). Continuous variables are expressed as mean and standard deviation. ^a^ Mann–Whitney U test. ^b^ Pearson’s chi-square test. ^c^ Fisher’s exact test.

**Table 3:** Burnout and absenteeism in nursing staff according to gender.

Variable	General Value (*n* = 108)	Male (*n* = 58)	Female (*n* = 96)	*p*
Burnout level	58.9 (±18.3)	56.5 (±17.4)	60.3 (±18.8)	0.184 ^d^
Burnout	108 (70.1)	39 (36.1)	69 (63.9)	0.543 ^b^
Dimensions				
Emotional exhaustion	21.4 (±12.1)	19.4 (±11.0)	22.7 (±12.6)	0.101 ^a^
Mild	67 (43.5)	29 (26.9)	38 (35.2)	0.206 ^b^
Moderate	42 (27.3)	16 (14.8)	26 (24.1)	0.946 ^b^
Severe	45 (29.2)	13 (12.0)	32 (29.6)	0.149 ^b^
Depersonalization	6.6 (±6.2)	6.0 (±5.8)	6.9 (±6.4)	0.444 ^a^
Mild	74 (48.1)	30 (27.8)	44 (45.8)	0.478 ^b^
Moderate	33 (21.4)	13 (12.0)	20 (18.5)	0.817 ^b^
Severe	47 (30.5)	15 (13.9)	32 (29.6)	0.329 ^b^
Personal accomplishment	30.7 (±8.7)	31.0 (±8.2)	30.5 (±9.0)	0.936 ^a^
Mild	84 (54.4)	5 (4.6)	7 (6.5)	0.765 ^c^
Moderate	45 (29.2)	23 (21.3)	35 (32.4)	0.692 ^b^
Severe	25 (16.2)	30 (27.8)	54 (50.0)	0.585 ^b^
Dimensional impact of burnout				
None	46 (29.9)	19 (12.3)	27 (17.5)	0.543 ^b^
One dimension	56 (36.4)	23 (14.9)	33 (21.4)	0.509 ^b^
Two dimensions	36 (23.4)	13 (8.4)	23 (14.9)	0.826 ^b^
Three dimensions	16 (10.4)	3 (1.9)	13 (13.5)	0.099 ^b^
Job tenure	129.7 (±88.9)	0.490 ^a^	128.4 (±91.4)	0.179 ^a^

Note. The percentages for the gender subcategories (male and female) were calculated using the total number of participants of each sex as the denominator (*n* = 58 and *n* = 96, respectively) to allow for proportional comparison between groups. Categorical variables are expressed as frequency (*n*) and percentage (%). Continuous variables are expressed as mean and standard deviation. ^a^ Mann–Whitney U test. ^b^ Pearson’s chi-square test. ^c^ Fisher’s exact test. ^d^ Student’s *t*-test.

**Table 4:** Determining factors associated with absenteeism in nursing staff.

Variable	General Value (*n* = 154)	Male (*n* = 58)	Female (*n* = 96)	*p*
Absence	81 (52.6)	36 (44.4)	45 (55.6)	0.067 ^b^
Absence	11 (7.1)	4 (4.9)	7 (8.6)	1.000 ^c^
Tardiness	44 (28.5)	15 (18.5)	29 (35.8)	0.563 ^b^
Early release	51 (33.1)	22 (27.2)	29 (35.8)	0.324 ^b^
Absence				
Uncertified sick leave	5 (45.4)	2 (20.0)	3 (60.0)	1.000 ^c^
Financial matter	0 (0.0)	0 (0.0)	0 (0.0)	-
Other job	2 (18.1)	2 (100.0)	0 (0.0)	0.140 ^c^
Transportation matter	0 (0.0)	0 (0.0)	0 (0.0)	-
Care of a dependent relative	0 (0.0)	0 (0.0)	0 (0.0)	-
Childcare	1 (9.0)	0 (0.0)	1 (100.0)	1.000 ^c^
Family matter	3 (27.2)	0 (0.0)	3 (100.0)	0.291 ^c^
Death of a relative	0 (0.0)	0 (0.0)	0 (0.0)	-
Victim of violence	0 (0.0)	0 (0.0)	0 (0.0)	-
Tardiness				
Uncertified sick leave	4 (9.0)	0 (0.0)	4 (100.0)	0.298 ^c^
Financial matter	2 (4.5)	2 (100.0)	0 (0.0)	0.140 ^c^
Other job	10 (22.7)	7 (70.0)	3 (30.0)	**0.042 ^c^**
Transportation matter	15 (34.0)	5 (33.3)	10 (66.4)	0.716 ^b^
Care of a dependent relative	5 (11.3)	0 (0.0)	5 (100.0)	0.157 ^c^
Childcare	4 (9.0)	0 (0.0)	4 (100.0)	0.298 ^c^
Family matter	4 (9.0)	1 (25.0)	3 (75.0)	1.000 ^c^
Death of a relative	0 (0.0)	0 (0.0)	0 (0.0)	-
Victim of violence	1 (2.2)	0 (0.0)	1 (100.0)	1.000 ^c^
Early release				
Uncertified illness	5 (9.8)	1 (20.0)	4 (60.0)	0.651 ^c^
Financial matter	3 (5.8)	1 (33.3)	2 (66.4)	1.000 ^c^
Other job	11 (21.5)	9 (81.8)	2 (18.2)	**0.003** ^c^
Transportation matter	6 (11.7)	3 (50.0)	3 (50.0)	0.673 ^c^
Care of a dependent relative	6 (11.7)	0 (0.0)	6 (100.0)	0.084 ^c^
Childcare	8 (15.6)	2 (25.0)	6 (75.0)	0.711 ^c^
Family matter	14 (27.4)	7 (50.0)	7 (50.0)	0.318 ^c^
Death of a relative	0 (0.0)	0 (0.0)	0 (0.0)	-
Victim of violence	0 (0.0)	0 (0.0)	0 (0.0)	-
Job tenure	129.7 (±88.9)	0.490 ^a^	128.4 (±91.4)	0.179 ^a^

Note. The percentages for the gender subcategories (male and female) were calculated using the total number of participants of each sex as the denominator (*n* = 58 and *n* = 96, respectively) to allow for proportional comparison between groups. Categorical variables are expressed as frequency (*n*) and percentage (%). Continuous variables are expressed as mean and standard deviation. ^a^ Mann–Whitney U test. ^b^ Pearson’s chi-square test. ^c^ Fisher’s exact test. Significant values in bold (*p* < 0.05).

## Data Availability

The data presented in this study are available on request from the corresponding author due to privacy and ethical restrictions.

## Public Involvement Statement

No public involvement in any aspect of this research.

## Guidelines and Standards Statement

This manuscript was written according to the STROBE guidelines for observational studies. A complete list of reporting guidelines can be accessed via the equator network: https://www.equator-network.org/ (accessed on 12 December 2025).

## Abbreviations

The following abbreviations are used in this manuscript:

CUCS	Centro Universitario de Ciencias de la Salud
IMSS	Instituto Mexicano del Seguro Social
CIBO	Centro de Investigación Biomédica de Occidente
EE	Emotional Exhaustion
DE	Depersonalization
PA	Personal Accomplishment
WHO	World Health Organization
ICD-11	International Classification of Diseases, 11th Revision
MBI	Maslach Burnout Inventory
DM	Diabetes Mellitus
HGZ	Hospital General de Zona
SPPSTIMSS	Servicios de Prevención y Promoción de la Salud para Trabajadores IMSS
